# Probe-Based Confocal Laser Endomicroscopy During Transurethral Resection of Bladder Tumors Improves the Diagnostic Accuracy and Therapeutic Efficacy

**DOI:** 10.1245/s10434-019-07200-6

**Published:** 2019-02-04

**Authors:** Jongsoo Lee, Seong Uk Jeh, Dong Hoon Koh, Doo Yong Chung, Min Seok Kim, Hyeok Jun Goh, Joo Yong Lee, Young Deuk Choi

**Affiliations:** 1 Department of Urology, Severance Hospital, Urological Science Institute, Yonsei University College of Medicine, Seoul, Korea; 2 Department of Urology, Gyeong-Sang National University College of Medicine, Jinju, Korea

## Abstract

**Purpose:**

This study was designed to assess the diagnostic accuracy and therapeutic efficacy of probe-based confocal laser endomicroscopy (pCLE), which provides real-time, in vivo histological information during transurethral resection of bladder tumors.

**Methods:**

We performed a prospective study between August 2013 and August 2014. pCLE was performed on a total of 119 lesions in 75 patients. We analyzed the diagnostic accuracy of pCLE by comparing the confocal image reports with the pathology reports of surgical specimen. Confocal images were interpreted by a single urologist blinded to the pathology reports. The therapeutic efficacy was analyzed by comparing the outcomes in pCLE and non-pCLE groups.

**Results:**

In a total of 119 lesions, 23 were benign and 96 were malignant. The detection accuracy for malignant lesions with pCLE was determined with a sensitivity and a positive predictive value (PPV) of 91.7% and 93.6%, respectively. For high-grade versus low-grade bladder cancer, sensitivity and PPV of pCLE were 94.5% and 89.7%, respectively. Distinguishing carcinoma in situ from inflammatory lesions also was accurate with sensitivity, specificity, and PPV of 71.4%, 81.3%, and 83.3%, respectively. The Kaplan–Meier curves revealed that the recurrence-free survival rate was significantly higher in the pCLE group than in the non-pCLE group (*p* = 0.031).

**Conclusions:**

Probe-based confocal laser endomicroscopy is a promising method to provide the surgeon during the transurethral resection of a bladder tumor with real-time tumor histology, regardless of the tumor’s gross appearance. Furthermore, it also may improve the therapeutic efficacy with longer recurrence-free periods.

Bladder cancer is one of the most common malignant tumors in the urinary tract.^1^ For newly diagnosed tumors, approximately 75% are nonmuscle-invasive bladder cancers (NMIBC), which have a high risk of recurrence, but a good survival rate.^2^ Recurrence and progression are more frequent in high-grade tumors.^3^–^5^ Hence, identification of high-grade tumors is crucial for the management of bladder cancer.

Although there is a clear consensus that carcinoma in situ (CIS) contribute to greater mortality and morbidity, there still are no favorable imaging techniques for their detection. In current everyday practice, the detection of CIS only depends on the findings from white-light cystoscopy (WLC) and biopsies. Although the biopsy is an accurate and widely accepted method for assessment, it may be unnecessary for treatment selection and carries the risk of complications. However, during an inspection of the bladder under WLC alone, the margin of a CIS is ambiguous, which interferes with the complete endoscopic tumor resection or may result in excessive cauterization of the bladder mucosa, possibly leading to voiding difficulties.

Recently, efforts for the development of optical imaging technologies were made to overcome the limitations of conventional WLC. The newly introduced narrow-band imaging (NBI) and blue-light cystoscopy (BLC) also are based on gross images only. However, probe-based confocal laser endomicroscopy (pCLE), based on histological findings and providing real-time histopathological information to the surgeon, is a more promising technique for both diagnostic and therapeutic purposes. Confocal laser endomicroscopy (CLE) was first introduced in 2004, and its usage has initially been expanded into the treatment of gastrointestinal and, later, biliopancreatic diseases. Nowadays, CLE is used in various specialties, such as pulmonology, dermatology, and urology.^6^–^10^

The pCLE system uses a semiflexible probe, which can be advanced to the bladder through the working channel of the cystoscope. The pCLE image is obtained by focusing low-energy laser light on a specific tissue layer that will emit fluorescent light, which is recorded by a photodetector and transformed into a gray-scale image that represents the specific tissue layer. The pCLE can provide a real-time intraoperative diagnosis of the tumor grade without the need for frozen biopsies.^11^,^12^

We hypothesized that structural differences of abnormal lesions in pCLE findings are highly correlated with abnormal histopathological findings in bladder lesions. Because of this reason, we also hypothesized that more precise TURB can be performed according to each pCLE findings which will maximize the therapeutic efficacy. To our knowledge, until now there were no prospective studies that assessed the diagnostic accuracy of pCLE in bladder cancer with only one study evaluating pCLE as a diagnostic tool. Therefore, we performed a prospective study to assess the diagnostic accuracy and therapeutic efficacy of pCLE for bladder cancer.

## Materials and Methods

### Patients

Between August 2013 and August 2014, pCLEs were performed on a total of 119 lesions in 75 patients. All patients who were planned for transurethral resection of bladder tumor (TUR-BT) were included in the pCLE group. Patients were excluded and placed into the non-pCLE group under the following conditions: inability to receive informed consent; fluorescein allergy; serum creatinine above the upper limit; pregnancy or breastfeeding; uncorrected coagulopathy; and severe thrombocytopenia. All patients provided written, informed consent before enrollment into the current study, which was approved by the Yonsei University Hospital Institutional Review Board (1-2010-0013).

### Probe-Based CLE

TUR-BT and pCLE image acquisitions were performed under spinal or general anesthesia and were completed by a single surgeon (YDC). Initially, we characterized the location, shape, and size of a lesion with conventional WLC. Before pCLE scanning, 300–400 mL of normal saline with 0.1% fluorescein (Novartis, Lake Forest, IL) was filled into the bladder cavity for tissue contrast. Five minutes after the fluorescein injection, pCLE images were obtained using a 2.5-mm diameter probe (UHD GastroFlex, CellVizio; Mauna Kea Technologies, Paris, France). This probe has a resolution of 1 µm, a field of view of 240 µm, and a 1000× magnification with 12 frames/s. To obtain diagnostic images, the probe was placed on the target lesion and scanned with pCLE. The mucosal tissue surrounding the target lesion also was scanned. After obtaining the images, monopolar TUR-BT was performed. The resected specimens were sent to the same pathologist at the same institution for histological review.

### Diagnostic Outcome Measures

Postoperatively, pCLE images were reviewed and classified into six categories (inflammation, papilloma, papillary urothelial neoplasm of low malignant potential [PUNLMP], low-grade tumor, high-grade tumor, and carcinoma in situ) by a single urologist who has been educated for classifying pCLE images. This reviewer was blinded to the medical histories and histological diagnoses of the patients. The histological diagnosis of each patient was used as a reference to assess the diagnostic accuracy of pCLE images through sensitivity, specificity, positive predictive value (PPV), and negative predictive value (NPV). To compare the diagnoses from the pCLE images with those from the histopathology report, we analyzed the 2 × 2 crosstab transformations malignant versus benign lesions, high-grade versus low-grade tumors, and CIS versus inflammation.

### Evaluation of the Therapeutic Effects of pCLE

The therapeutic efficacy of pCLE was evaluated only among patients who had T1 high-grade urothelial carcinoma. All information was obtained prospectively, and comparisons were made between pCLE and non-pCLE groups. We resected all masses and cauterized any grossly suspicious area. All patients with a pathologic diagnosis of T1 high-grade urothelial carcinoma received six courses of adjuvant intravesical bacillus Calmette-Guérin therapy. Follow-up cystoscopy and cytology were done in regular intervals of 3 months for 2 years and additionally at times of gross hematuria events. Recurrence was defined as a cystoscopic and pathologic tumor detection.

### Statistical Analysis

Qualitative data are presented as frequencies or percentages; continuous data are provided as means and standard deviation without comparing each group. Sensitivity, specificity, PPV, and NPV with 95% confidence intervals were calculated with Medcalc (version 18.3.; Mariakerke, Belgium). The Kaplan–Meier curve was extracted by SPSS version 23 (SPSS Inc., Chicago, IL). Associations were considered statistically significant for two-sided *p* < 0.05.

## Results

Patient demographics and lesion characteristics are summarized in Table 1. A total of 75 patients were eligible to be enrolled in the study, which included 119 lesions. Male to female ratio was 6.5:1 with a total mean age of 68.32 ± 9.45 years. Histopathological results established that 23 (19.3%) of the lesions were benign (papilloma, 4; inflammation, 16; low malignant potential, 3) and 96 (80.7%) of the lesions were malignant (low-grade urothelial carcinoma, 20; high-grade urothelial carcinoma, 55; carcinoma in situ, 21). Based on gross appearance, 78 lesions were papillary (65.5%) and 41 lesions were flat (34.5%). The pCLE was performed successfully without any adverse effects in all pCLE group patients. Also, there were no systemic toxicity, allergic, or hypersensitive reactions with intravesical fluorescein administration.

**Table 1:** Patient demographics and lesion characteristics

Demographic	Total	Lesions					
		Benign			Malignant		
		Inflammation	Papilloma	Low malignant potential	Low grade	High grade	CIS
Lesions, no.	119	16	4	3	20	55	21
Sex, no.							
Male	90	9	3	2	16	44	16
Female	29	7	1	1	4	11	5
Age	68.32	67.55	68.00	44.00	68.00	69.89	67.64
(mean ± SD)	± 9.45	± 7.41	± 15.56	± 5.20	± 8.08	± 9.39	± 10.49
Histopathological TNM staging							
T0	23	16	4	3	0	0	0
Ta	37	0	0	0	20	17	0
Tis	21	0	0	0	0	0	21
T1	28	0	0	0	0	28	0
T2a	9	0	0	0	0	9	0
T2b	0	0	0	0	0	0	0
T3a	1	0	0	0	0	1	0
T3b	0	0	0	0	0	0	0
T4	0	0	0	0	0	0	0
Shape							
Nonpapillary	41	16	0	0	0	4	21
Papillary	78	0	4	3	20	51	0

*CIS* carcinoma in situ, *SD* standard deviation

### pCLE Image Outcomes

**Fig. 1:**
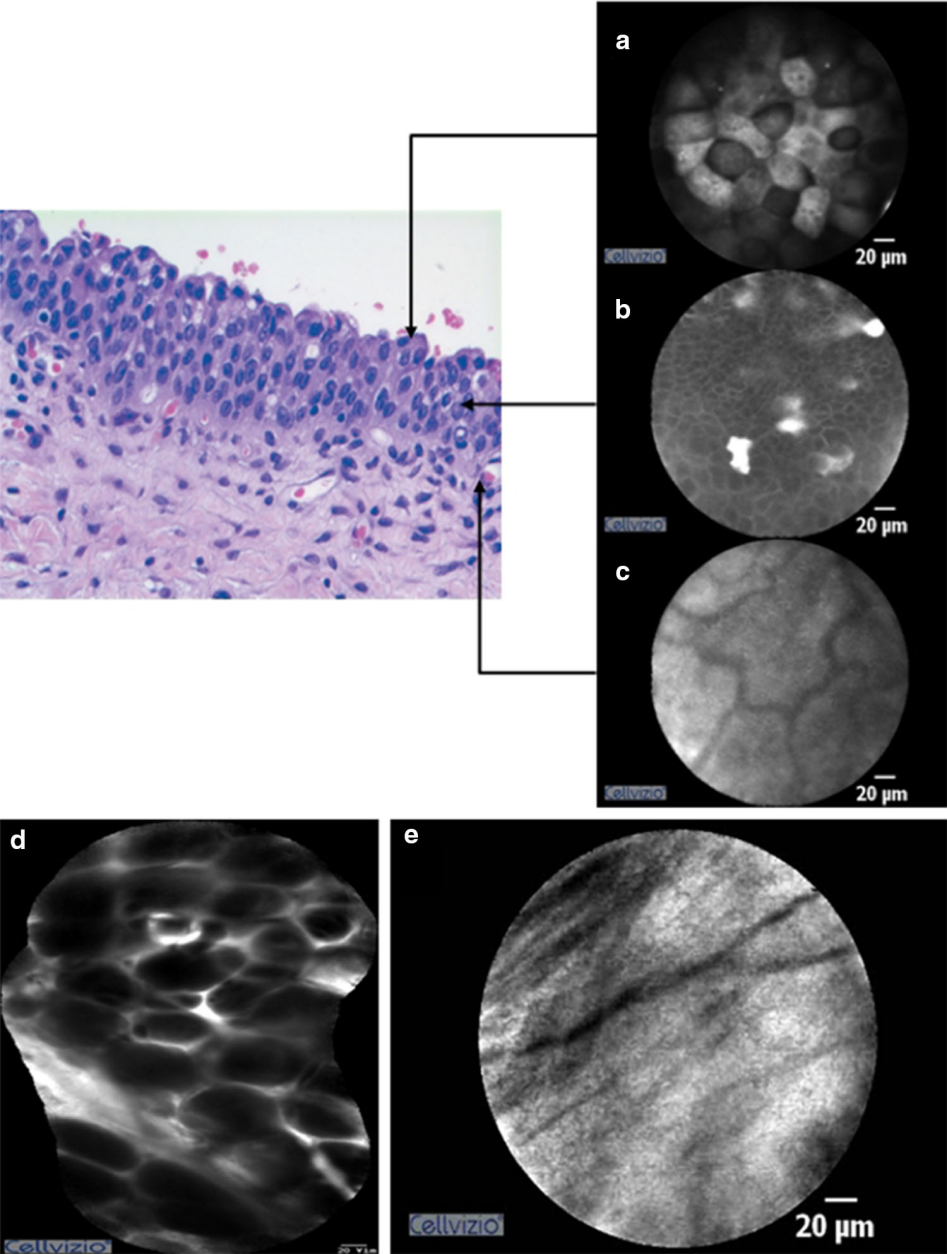
Pathology pictures and probe-based confocal laser endomicroscopy (pCLE) images of the normal bladder (**a-c**) and the resection bed after TUR-BT (**d**, **e**). **a** Umbrella cells; **b** intermediate cells; **c** lamina propria; **d** fat tissue; and **e** muscularis propria

**Fig. 2:**
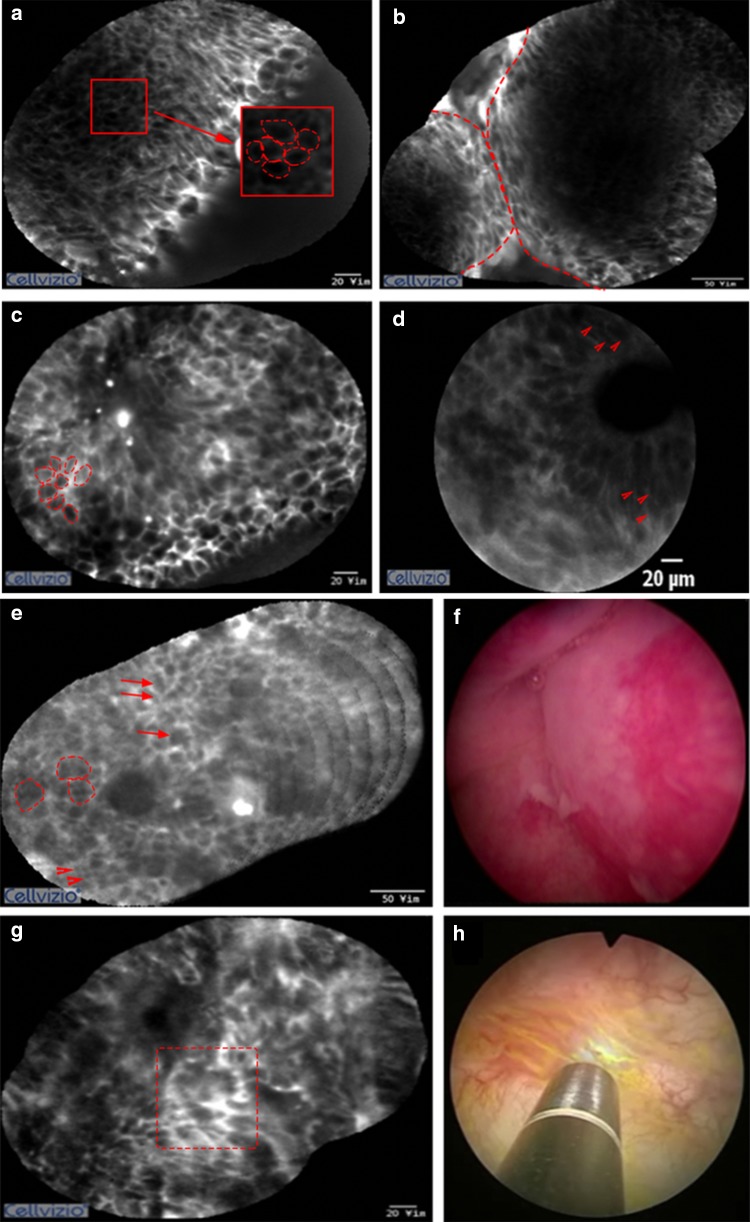
Probe-based confocal laser endomicroscopy (pCLE) images. **a**, **b** Low-grade tumor showing monomorphic cohesive cells (dashed circle) with distinct borders (dash line) and a well-organized papillary architecture. **c**, **d** High-grade tumor displaying pleomorphic noncohesive cells with indistinct borders and disorganized papillary architecture (arrowheads). **e**, **f** Inflammatory lesion showing small monomorphic cells (arrowheads) with small cells arranged in clusters (dashed circle) and loosely infiltrating cells with distinct cell border in the lamina propria (arrows). **g**, **h** Carcinoma in situ presenting a flat disorganized architecture with pleomorphic and noncohesive cells with an indistinct border

The pCLE was performed on the normal mucosa of the bladder for all patients in the pCLE group. Normal bladder mucosa of pCLE yielded images of large umbrella cells and uniform intermediate cells on the surface. A lamina propria with its vascular network and scarce cellular connective tissue also were identified. On the resection bed after TUR-BT, the fat tissue and muscularis propria were occasionally visualized by pCLE (Fig. 1). Using only pCLE, it was difficult to differentiate between papilloma and PUNLMP. Both lesions showed thin, papillary, fibrovascular stalks and no other significant differences in cell size and shape. However, in most cases, confocal images revealed definite differences between normal mucosa, low-, and high-grade urothelial carcinoma. In low-grade carcinoma, branched-papillary fibrovascular stalks and densely packed monomorphic urothelial cells were noted. Some of these confocal diagnostic criteria showed an overlap between high- and low-grade urothelial carcinoma, such as the presence of fibrovascular stalks or densely packed cells. High-grade urothelial carcinoma, however, exhibited more loss of cellular cohesiveness of pleomorphic cells. Among flat lesions, CISs exhibited more pleomorphic and large cells compared with inflammatory lesions as well as extensive acellular areas and indistinct cell borders (Fig. 2).

### Diagnostic Accuracy of pCLE

Our comparison of data from pCLE images and histopathological reports is summarized in Table 2. The data analysis revealed that the overall rate for diagnosis of malignant to benign lesions with pCLE alone has a sensitivity of 91.7% (95% confidence interval [CI], 84.2–96.3), a specificity of 73.9% (95% CI 51.6–89.8), a PPV of 93.6% (95% CI 88–96.7), and an NPV of 68.0% (95% CI 51.2–81.2). The overall sensitivity for the diagnosis of high-grade versus low-grade papillary tumors was 94.5% (95% CI 84.9–98.7). Its specificity was 66.7% (95% CI 41.0–86.7), the PPV was 89.7% (95% CI 81.8–94.4), and the NPV was 80.0% (95% CI 55.9–92.7). The overall sensitivity for the diagnosis of CIS versus inflammation was 71.4% (95% CI 47.8–88.7), the specificity was 81.3% (95% CI 54.4–96.0), the PPV was 83.3% (95% CI 63.5–93.5), and the NPV was 68.4% (95% CI 51.4–81.6).

**Table 2:** Comparison of histological diagnosis versus pCLE diagnosis

Histopathological diagnosis	pCLE diagnosis						Total
	Inflammation	Papilloma	Low malignant potential	Low-grade tumor	High-grade tumor	CIS	
Inflammation	13	0	0	0	0	3	16
Papilloma	0	4	0	0	0	0	4
Low malignant	0	0	0	3	0	0	3
potential							
Low-grade	0	1	1	12	6	0	20
tumor							
High-grade	0	0	0	3	52	0	55
tumor							
CIS	6	0	0	0	0	15	21
Total	19	5	1	18	58	18	119

*pCLE* probe-based confocal laser endomicroscopy, *CIS* carcinoma in situ

### Therapeutic Effects of pCLE

**Fig. 3:**
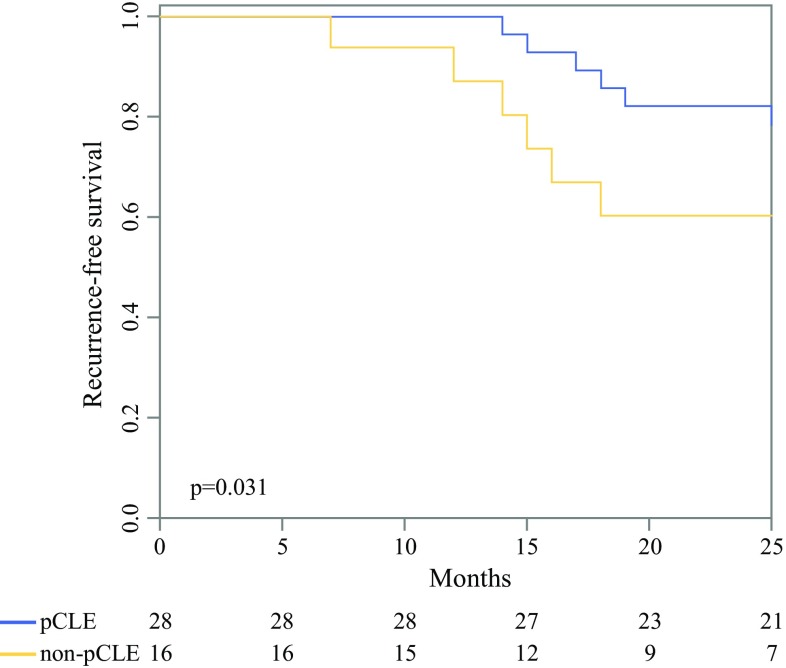
Kaplan–Meier estimate between pCLE and non-pCLE groups. Recurrence-free survival is significantly higher in the pCLE group than in the non-pCLE group (*p* = 0.031)

In pCLE and non-pCLE groups, there were 28 and 16 lesions of T1 high-grade urothelial carcinoma, respectively, which were analyzed to evaluate the therapeutic effects of pCLE. The median follow-up time was 32.5 (interquartile range [IQR], 18.75–37.00) months. The recurrence-free survival rate in 30 months was 78.2% (95% CI 59.6–92.8) in the pCLE group and 60.3% (95% CI 40.0–90.9) in the non-pCLE group. The recurrence-free survival was significantly longer in the pCLE group than in the non-pCLE group (Fig. 3).

## Discussion

The standard procedure to diagnose and confirm bladder cancer is TUR-BT performed under visualization with conventional WLC. However, WLC has several well-recognized shortcomings. Due to its dependence on visual appearance, WLC sometimes can overlook bladder cancer, especially flat or subtle papillary lesions and CIS lesions that appear as normal mucosa. This increases the chances for an incomplete resection or positive surgical margins.

Jancke et al.^13^ reported that incomplete TUR-BT is a common problem. Their study revealed that 26% of patients had positive margins or residual tumors following TUR-BT, which may be a risk factor for cancer recurrence. Another limitation of WLC is that it is not useful in predicting specific tumor grades or invasion depth, factors that influence the oncological outcomes of bladder cancer.

The limitations of conventional WLC have prompted the development of a new generation of optical technologies, such as NBI and BLC.^14^–^17^ Nevertheless, these technologies are limited to gross or macroscopic views and also have demonstrated high false-positive detection rates.^18^,^19^ By contrast, pCLE offers real-time, in vivo histological assessment, without the need for biopsies.^20^ In addition, pCLE probes have a diverse range in sizes, from 0.9 to 2.5 mm, which can be fitted through the working channel of both rigid and flexible cystoscopes.

Despite the potential usefulness of pCLE for bladder cancer diagnosis, there is a lack of literature regarding its efficacy. To our knowledge, until now only one study reported the accuracy of pCLE in the diagnosis of bladder cancer. Chang et al. evaluated the interobserver agreement and diagnostic accuracy of pCLE for bladder cancer, and they concluded that pCLE may have advantages compared with WLC alone.^21^ However, compared with our study, their study noted a relatively low specificity and sensitivity for pCLE. Our study demonstrates high accuracy rates between histological diagnosis and confocal images regardless of tumor appearance and grade. These findings are consistent with previous studies that have evaluated the diagnostic role of pCLE in other organs.

In gastroenterology, Pohl et al.^22^ evaluated 296 biopsy sites from 38 patients for high-grade dysplasia and early carcinoma in Barrett’s esophagus patients to assess the preliminary accuracy of pCLE. They report an overall accuracy of 88% to 93%. Similarly, Dunbar et al. achieved an accuracy of 97.8% for the diagnosis of chronic ulcerative colitis with an integrated endoscope-based CLE system.^23^

Recurrence and progression are more frequent in high-grade bladder cancer.^3^–^5^ In our study, a sensitivity and a PPV of 94.5% and 89.7%, respectively, were determined for the pCLE-based differentiation between high-grade and low-grade papillary tumors. Thus, pCLE is able to detect high-grade cancers with high accuracy. When a high-grade cancer was suspected, the operator had more evidence and confidence to perform an adequately deep resection. This change in the operative method may improve the oncological outcome, and the bladder-preserving period can be prolonged.

Our study also analyzed the therapeutic effect of pCLE in T1 high-grade cancer. We found that within 30 months, the recurrence-free rate was higher in patients who underwent TUR-BT under pCLE than in the non-pCLE group with 78.2% and 60.3%, respectively. The Kaplan–Meier curve also shows a higher recurrence-free survival rate for the pCLE group. Therefore, we conclude that pCLE has therapeutic effects in addition to its diagnostic purposes.

Additionally, pCLE was useful to differentiate flat lesions, such as CIS and inflammation. It is known that CIS has a high risk of tumor recurrence and progression. Failure to identify CIS lesions may result in a worse prognosis. Geavlete et al.^24^ reported that the CIS detection rate of WLC is only 63%. In our study, however, the detection rate of CIS by pCLE was 83.3%, which is almost 20% higher than that by WLC alone. Therefore, pCLE may be a good tool for an accurate diagnosis when CIS lesions hardly can be distinguished clinically from a bladder inflammation.

There are many methods for enhancing the diagnostic accuracy and therapeutic efficacy for bladder cancer. These include fluorescence, BLC, NBI, molecular imaging, optical coherence tomography (OCT), and CLE. NBI and BLC are used in clinical practice, but there are limitations to what surgeons can discern from the relatively subjective gross images. One of the most effective techniques to distinguish abnormal lesions in real-time with high accuracy in the urological field seems to be pCLE. By providing real-time histological images, it is a highly appropriate procedure, which can be performed in the operating room. Our study reveals that pCLE is one of the promising methods that increase the detection rate of malignant lesions, and the use of pCLE can aid in a more complete resection of bladder cancer. It also may reduce the tumor recurrence rate compared with TUR-BT done under conventional WLC alone.

Despite the promising results reported in this study, there are several limitations. First, although it is a prospective study, there may be a bias, because pCLE and non-pCLE groups are not completely randomized. Second, to compare therapeutic effects of pCLE, only T1 high-grade cases were selected, which have to be expanded to other stages in the near future. Third, the operation was performed by a single surgeon with an experience of a few thousand TUR-BTs. Surgeons with less experience may not reproduce our study results when they start performing pCLEs. Fourth, the pCLE images were interpreted by a single urology specialist; any inter-researcher variability has not been considered in this setting. Fifth, the number of lesions included in the study is small. Therefore, multicenter studies with a larger cohort are needed.

## Conclusions

Probe-based confocal laser endomicroscopy is a promising method to detect malignant lesions of the bladder regardless of tumor appearance. It has a high accuracy of differentiating CIS, low-, and high-grade urothelial carcinomas with additional therapeutic efficacy by providing in real-time the surgeon with a tumor histology. This has the potential to affect standard TUR-BTs and may prompt the use of more appropriate procedures, thus leading to longer recurrence-free periods.

## References

[1] Lynch CF, Cohen MB (1995). Urinary system. Cancer.

[2] Babjuk M, Oosterlinck W, Sylvester R, Kaasinen E, Böhle A, Palou-Redorta J (2008). EAU guidelines on non-muscle-invasive urothelial carcinoma of the bladder. Eur Urol.

[3] Brausi MA (2013). Primary prevention and early detection of bladder cancer: two main goals for urologists. Eur Urol..

[4] Jakse G, Loidl W, Seeber G, Hofstädter F (1987). Stage T1, grade 3 transitional cell carcinoma of the bladder: an unfavorable tumor?. J Urol.

[5] Heney NM, Ahmed S, Flanagan MJ (1983). Superficial bladder cancer: progression and recurrence. J Urol.

[6] Wiesner C, Jäger W, Salzer A (2011). Confocal laser endomicroscopy for the diagnosis of urothelial bladder neoplasia: a technology of the future?. BJU Int.

[7] Kiesslich R, Burg J, Vieth M (2004). Confocal laser endoscopy for diagnosing intraepithelial neoplasias and colorectal cancer in vivo. Gastroenterology.

[8] Langley RG, Walsh N, Sutherland AE (2007). The diagnostic accuracy of in vivo confocal scanning laser microscopy compared to dermoscopy of benign and malignant melanocytic lesions: a prospective study. Dermatology.

[9] Fuchs FS, Zirlik S, Hildner K (2011). Fluorescein-aided confocal laser endomicroscopy of the lung. Respiration.

[10] Wallace M, Lauwers GY, Chen Y (2011). Miami classification for probe-based confocal laser endomicroscopy. Endoscopy.

[11] Lopez A, Liao JC (2014). Emerging endoscopic imaging technologies for bladder cancer detection. Curr Urol Rep.

[12] Sylvester RJ, van der Meijden AP, Oosterlinck W (2006). Predicting recurrence and progression in individual patients with stage Ta T1 bladder cancer using EORTC risk tables: a combined analysis of 2596 patients from seven EORTC trials. Eur Urol.

[13] Jancke G, Rosell J, Jahnson S (2012). Residual tumour in the marginal resection after a complete transurethral resection is associated with local recurrence in Ta/T1 urinary bladder cancer. Scand J Urol Nephrol..

[14] Cauberg EC, Kloen S, Visser M (2010). Narrow band imaging cystoscopy improves the detection of non–muscle-invasive bladder cancer. Urology..

[15] Herr H, Donat M, Dalbagni G, Taylor J (2010). Narrow-band imaging cystoscopy to evaluate bladder tumours–individual surgeon variability. BJU Int.

[16] Cauberg EC, de Bruin DM, Faber DJ, van Leeuwen TG, de la Rosette JJ, de Reijke TM (2009). A new generation of optical diagnostics for bladder cancer: technology, diagnostic accuracy, and future applications. Eur Urol.

[17] Jocham D, Stepp H, Waidelich R (2008). Photodynamic diagnosis in urology: state-of-the-art. Eur Urol..

[18] Schmidbauer J, Remzi M, Klatte T (2009). Fluorescence cystoscopy with high-resolution optical coherence tomography imaging as an adjunct reduces false-positive findings in the diagnosis of urothelial carcinoma of the bladder. Eur Urol.

[19] Ray ER, Chatterton K, Khan MS, Thomas K, Chandra A, O’Brien TS (2009). Hexylaminolaevulinate “blue light” fluorescence cystoscopy in the investigation of clinically unconfirmed positive urine cytology. BJU Int.

[20] Ray ER, Chatterton K, Thomas K, Khan MS, Chandra A, O’Brien TS (2009). Hexylaminolevulinate photodynamic diagnosis for multifocal recurrent nonmuscle invasive bladder cancer. J Endourol..

[21] Chang TC, Liu J-J, Hsiao ST (2013). Interobserver agreement of confocal laser endomicroscopy for bladder cancer. J Endourol.

[22] Pohl H, Rosch T, Vieth M (2008). Miniprobe confocal laser microscopy for the detection of invisible neoplasia in patients with Barrett’s esophagus. Gut.

[23] Dunbar KB, Okolo P, Montgomery E, Canto MI (2009). Confocal laser endomicroscopy in Barrett’s esophagus and endoscopically inapparent Barrett’s neoplasia: a prospective, randomized, double-blind, controlled, crossover trial. Gastrointest Endosc..

[24] Geavlete B, Multescu R, Georgescu D, Jecu M, Stanescu F, Geavlete P (2012). Treatment changes and long-term recurrence rates after hexaminolevulinate (HAL) fluorescence cystoscopy: does it really make a difference in patients with non-muscle-invasive bladder cancer (NMIBC)?. BJU Int..

